# Polyelectrolyte-Complex-Based Hydrogel Inserts for Vaginal Delivery of Posaconazole and Probiotics

**DOI:** 10.3390/gels9110851

**Published:** 2023-10-27

**Authors:** Sanjeevani Deshkar, Purva Yeole, Jayashri Mahore, Ankita Shinde, Prabhanjan Giram

**Affiliations:** 1 Department of Pharmaceutics, Dr. D. Y. Patil Unitech Society’s, Dr. D. Y. Patil Institute of Pharmaceutical Science & Research, Pune 411018, India; purvayeole11@gmail.com (P.Y.); jayashri.topale@gmail.com (J.M.); 10ankita10@gmail.com (A.S.); 2 Department of Pharmaceutical Sciences, The State University of New York, Buffalo, NY 14214, USA

**Keywords:** vaginal candidiasis, polyelectrolyte complex, hydrogel insert, posaconazole, chitosan, probiotic, mucoadhesion, *Candida albicans*

## Abstract

Worldwide, 40 to 50% of women suffer from reproductive tract infections. Most of these infections are mixed infections, are recurrent and difficult to treat with antimicrobials or antifungals alone. For symptomatic relief of infections, oral antimicrobial therapy must be combined with topical therapy. The purpose of this work is to optimize and develop a polyelectrolyte complex (PEC) of chitosan/anion for the formulation of posaconazole- and probiotic-loaded vaginal hydrogel inserts with prolonged release and significant mucoadhesion. PECs were prepared using chitosan as cationic and carrageenan, pectin and polycarbophil as anionic polymers via a lyophilization technique. PEC formation was confirmed by scanning electron microscopy, Fourier transform infrared spectroscopy and differential scanning calorimetry, by observing changes in its surface, physical and thermal properties. The probiotic, *Lactobacillus casei*, was added to the PEC during the lyophilization process and the effect on the probiotic viability was studied. The PECs were further compressed along with posaconazole to form hydrogel inserts and optimized using a 3^2^ full-factorial design. The hydrogel inserts were assessed for swelling behavior, drug release, in vitro mucoadhesion and in vitro antifungal activity. The chitosan–pectin hydrogel insert demonstrated excellent mucoadhesion (1.25 N), sustained drug release (88.2 ± 2.4% in 8 h) and a swelling index of 154.7%. The efficacy of hydrogel inserts was evaluated using in vitro study with a co-culture of *Lactobacillus casei* and *Candida albicans*. This study revealed an increase in *Lactobacilli casei* count and a significant drop in the viable count of *Candida albicans* (4-log reduction in 24 h), indicating the effectiveness of hydrogel inserts in alleviating the fungal infection. Overall, our study demonstrated the potential of the hydrogel insert for preventing vaginal infection and restoring normal vaginal microbiota.

## 1. Introduction

Vaginal candidiasis (VC) is the second most common reproductive tract infection caused due to overgrowth of *Candida* spp. According to estimates, between 70 and 75% of women will experience VC at some point in their lifetime and about 50% of women have already had VC. According to reports, 5–8% of women experience recurrent VC and 40–50% of women may have a frequent VC infection [1]. Although there are several factors that cause VC, the most common are prolonged antibiotic use, hormonal fluctuations, immunocompromised conditions, menopause and diabetes. Treating patients with VC can be extremely challenging due to recurrence and resistance to antifungal agents [2].

Currently, the primary line of treatment for VC includes oral therapy with azoles such as fluconazole, which has many limitations such as cytochrome-P450-mediated drug interactions, fungal resistance and recurrence [3]. Moreover, oral therapies alone may not give symptomatic relief, thus an effective topical therapy is advisable. Clinically, it is established that topical therapy for vaginal infections is as efficient as oral therapy; in particular, second-generation anti-fungal triazoles have been reported to exhibit greater efficacy in studies carried out in vitro against a wide range of fungi [4].

Posaconazole (POS) is an effective second-generation antifungal triazole agent with a broad spectrum of activity against *Candida albicans*, *C. parapsilosis*, *C. glabrata*, *C. tropicalis*, *zygomycetes*, mucor and *Aspergillus species* [5]. It prevents the production of ergosterol, a vital component of the fungal cell wall, by inhibiting the fungal cytochrome P450 enzymes by attaching to and blocking lanosterol-14alpha-demethylase [6]. POS is effective clinically in highly invasive infections and immunocompromised patients and is able to suppress even mutant strains that are resistant to fluconazole, itraconazole and voriconazole [7]. It is presently available as an oral tablet or intravenous solution. POS has not been previously reported for vaginal use; however, clinical trials have demonstrated the efficacy of POS against mucosal candidiasis in patients with HIV infections, where it showed excellent tolerance and provided prolonged protection even after discontinuation of treatment [8].

The topical application of antifungal drugs is often limited due to their lower solubility, resulting in poor tissue uptake and availability. Additionally, the self-cleansing action of vagina reduces the retention of the drug in vaginal tract, further reducing its effectiveness [9]. Mucoadhesive polymers provide several benefits in vaginal drug delivery by prolonging contact time, targeting drug delivery, enhancing bioavailability, enabling controlled release and ensuring protection and stability [10]. Recently, mucoadhesive systems such as the polyelectrolyte complex (PEC) have shown potential in prolonging the residence time of vaginal systems and, consequently, sustaining the drug release [11]. The formation of PECs is facilitated by electrostatic interactions between oppositely charged polymers without the involvement of any chemical cross-linking agent. They can interact and form a complex due to the attractive forces between the charged groups. PECs can be formed by lyophilization in solution, as well as in the solid state [12]. Chitosan (CH) is an excellent mucoadhesive, biocompatible and biodegradable cationic polymer that has been widely studied for mucosal applications. However, it quickly disintegrates in an acidic environment and hence has less potential to prolong or control the release of drugs at this pH [13]. The formulation of PECs of chitosan with oppositely charged anionic polymers can address this issue. Darwesh et al. [14] optimized chitosan–alginate-PEC-based vaginal inserts of fluconazole. The inserts demonstrated effective antifungal activity in vitro and in vivo in a rat model, with significant reduction in the inflammation due to infection. Bigussi et al. [15] developed chlorhexidine vaginal inserts made from a polyelectrolyte complex of chitosan and carboxymethyl cellulose and reported their antifungal efficacy in vitro.

The aim of the current study was to optimize and develop the chitosan/anion-PEC-based hydrogel inserts for the vaginal delivery of POS with prolonged release and significant mucoadhesion. POS or its formulation for the vaginal route has not been previously reported. Therefore, in this study we are proposing a vaginal hydrogel formulation of POS in combination with a probiotic, *Lactobacilli casei*. *Lactobacilli* spp. are a critical component of the vaginal microbiome and their role in maintaining the vaginal health is well established [16]. In case of vaginal infections, there is imbalance of the vaginal flora and even if an antimicrobial therapy is given to suppress the infection, the lactobacilli in the flora are also compromised. This increases the chances of recurrence of infections [17]. Probiotics offer multiple benefits in the treatment and prevention of vaginal infections as adjuvants. They restore the natural vaginal microbiota, provide antimicrobial effects, modulate the immune response and provide protection against recurrence [18].

For this study, three anionic polymers, carrageenan (CA), pectin (PE) and polycarbophil (PC) were selected based on the literature. CA is an anionic mucoadhesive biopolymer reported for use in the vaginal cavity due to its antimicrobial activity. It has many advantages such as prolonged drug release, enhanced adhesion and localized drug delivery. It is naturally occurring, high molecular weight, non-irritating and non-toxic, making it suitable for use in biomedical applications [19]. PE is an anionic water-soluble biopolymer with repeating units of D-galacturonic acid. Based on the methoxylation of a carboxylic group, it is graded for different applications. It has been used as a mucoadhesive polymer in vaginal formulations owing to its biocompatibility, bioadhesion, biodegradability and low toxicity [20]. PC is a synthetic mucoadhesive polymer that is chemically cross-linked using divinyl glycol. It has been clinically explored for many vaginal formulations and has the ability to adhere to vaginal mucosa with sustained drug release. It is reported to provide a soothing and protective effect on the vaginal mucosa [21]. PECs of chitosan were formulated with CA, PE and PC by a lyophilization technique and PEC formulations were optimized via a 3^2^ full-factorial design. The formation of PECs was confirmed by scanning electron microscopy (SEM), Fourier transform infrared spectroscopy (FTIR) and differential scanning calorimetry (DSC). Lyophilized PECs were further used to formulate POS- and probiotic (*Lactobacillus casei*)-loaded hydrogel inserts for vaginal delivery. The developed inserts were evaluated for in vitro water uptake, drug release and antifungal assay using a co-culture technique.

## 2. Result and Discussion

### 2.1. Solid Dispersion of Posaconazole

POS is a BCS class II drug with very poor aqueous solubility. Solid dispersion (SD) formulation with a hydrophilic carrier is one of the effective approaches for improving the drug solubility of this class. Earlier reports have demonstrated a significant increase in the solubility of POS by formulating it into solid dispersion [22].

The solubility enhancement by hydrophilic carriers in SD could be attributed to conversion of the drug into an amorphous form, the molecular dispersion of the drug in the carrier matrix or wettability improvement via the carrier increasing solute–solvent interactions [23]. Kolliphor^®^ P 188 is a non-ionic surfactant polymer that is often used as a carrier for poorly water-soluble drugs. It is a tri-block copolymer consisting of a hydrophobic polypropylene (PPO) unit and a hydrophilic polyethylene (PEO) unit. It is used to formulate third-generation solid dispersions with improved stability owing to its ability to suppress drug precipitation from the formulated dispersions [24]. Since POS has tendency to precipitate in aqueous environments due to its very poor water solubility, a solid dispersion of POS was prepared with Kolliphor^®^ P 188 as carrier, considering its solubilizing and emulsifying potential.

**Figure 1:**
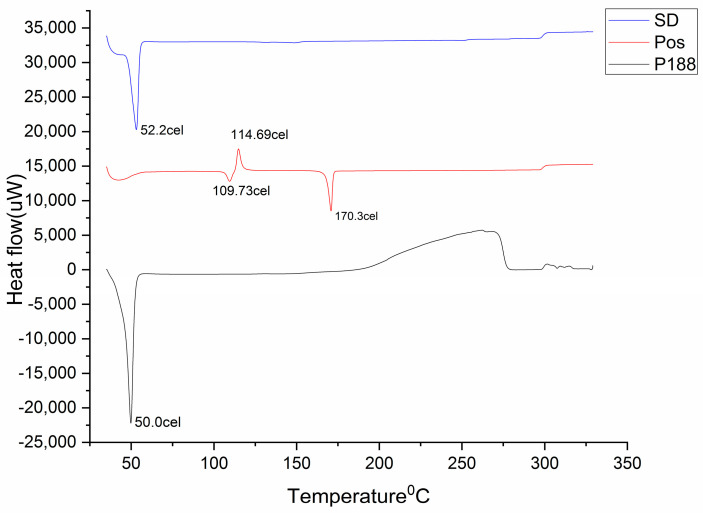
DSC of solid dispersion (SD), posaconazole (POS), Kolliphor^®^ P 188 (P188).

In order to confirm the formation of solid dispersion, DSC analysis of POS, Kolliphor^®^ P 188 and solid dispersion was performed (Figure 1). POS showed a sharp endothermic peak at 170.3 °C, indicating the melting point and crystalline nature of the POS. Kramarczyk et al. [25] described this endothermic peak as the melting event of POS polymorphic form I, which is anhydrous. A small peak before the melting of drug, at 109.7 °C, indicated nematic phase transition, which is characteristic of POS and other triazoles [26]. Kolliphor^®^ P 188 showed a sharp endothermic peak around 50.0 °C, indicating its melting point [27]. In the solid dispersion DSC curve, there was an endotherm at 52.2 °C represented melting of Kolliphor^®^ P 188. There was complete disappearance of the POS endotherm. Kramarczyk et al. [25] reported the depression in the melting point of a drug as indicative of the miscibility of drug and polymer in amorphous solid dispersion systems. The depression in the melting point is proportional to the amount of polymer used in the solid dispersions. If the polymers are used in high proportions in solid dispersions, DSC curves may result in the complete disappearance of the drug melting point. Thus, in the DSC curve of SD (Figure 1), the disappearance of the endothermic peak of POS confirmed the complete dispersion of the POS in carrier and the formation of solid dispersion [26].

### 2.2. Saturation Solubility

In order to study the impact of solid dispersion on POS solubility, a saturation solubility study was carried out. The saturation solubility of POS and solid dispersion in water were found to be 0.39 ± 0.23 mg/mL and 1.451 ± 0.18 mg/mL, respectively. In citrate buffer pH 4.8, the solubility was 0.96 ± 0.09 mg/mL and 1.885 ± 0.27 mg/mL, respectively. POS, as a triazole (pKa 4.6), has higher solubility in citrate buffer pH 4.8 compared with water. The solubility of solid dispersion was 1.95-fold more than the pure drug in citrate buffer pH 4.8 and 3.7-fold more in water. These results are in agreement with the work of Danda et al. [28] on amorphous solid dispersions of posaconazole. In this study, the equilibrium solubility of crystalline posaconazole was reported to be 0.026 mg/mL at pH 2, whereas the solubility of amorphous posaconazole was 0.137 mg/mL. When formulated as amorphous solid dispersions with carrier poly (1-vinylpyrrolidone-co-vinyl acetate), i.e., PVP/VA 64, the solubility of POS via a non-sink dissolution study in pH 2.0 was increased to 0.304 mg/mL (a 2.3-fold increase).

There are no reports of solid dispersions of POS with Kolliphor^®^ P 188. However, Kolliphor^®^ P 188 is known to increase the solubility of poorly soluble drugs by micellar solubilization. Being a surfactant-based polymer, it has ability to form a self-assembled micellar structure in solution above a critical concentration and critical temperature. The hydrophobic PPO units associate together to form an inner core of the micelle and a hydrophilic PEO unit is oriented towards the outer aqueous environment. The poorly soluble actives are entrapped and get solubilized into the lipophilic core, resulting in solubility enhancement [27]. Thus, the increase in the solubility of POS by Kolliphor^®^ P 188 SD could be attributed to this micellar solubilization.

### 2.3. Characterization of the Polyelectrolyte Complex

#### 2.3.1. FESEM

**Figure 2:**
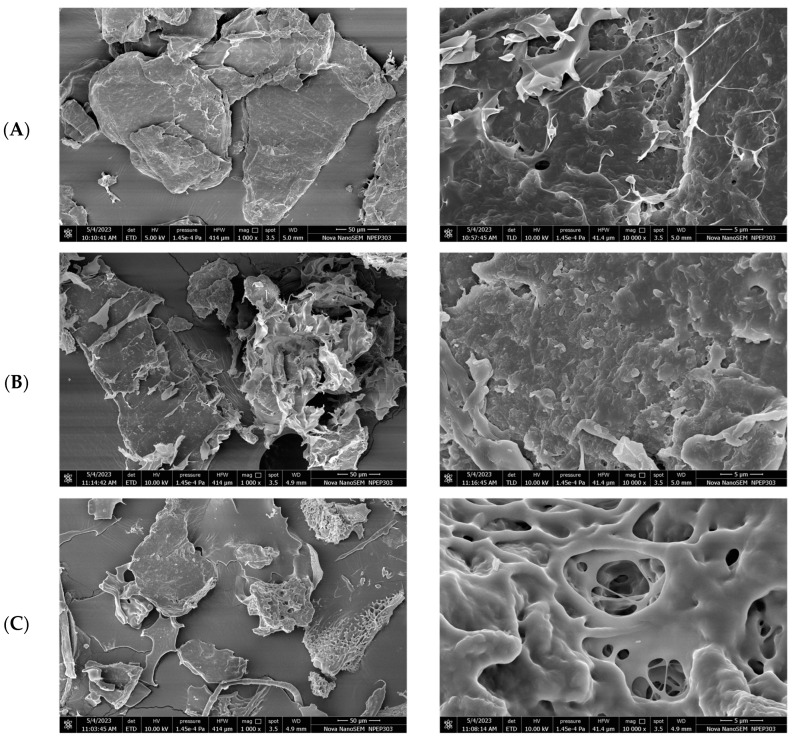
Scanning electron microscopy. (from top to bottom) (**A**) Chitosan–Pectin. (**B**) Chitosan–Carrageenan. (**C**) Chitosan–Polycarbophil (1000 × 10,000 magnification).

FESEM was used to study the surface morphology of the PEC samples (Figure 2). The differences in the surface morphology and microstructures of PECs of CH with PE, CA and PC may be correlated with their porosity and swelling ability. The lyophilized PECs at lower magnification appeared as large irregular shaped aggregates with a dense three-dimensional structure. At higher magnifications, the CH-PE PEC appeared as a matrix of fibrous threadlike structures made from interconnected particles. Similar to the report by Davydova et al. [29], the CH-CA PEC exhibited a denser structure with a rough surface and very small vacuoles within its structure. The CH-PC PEC showed a porous interconnected network structure with bigger vacuoles in it. Compared with the other PECs, the surface of the CH-PE PEC was more compact and less porous. A similar morphology for CH-PE PEC was reported by Costa et al. [30]. Scanning electron micrographs of CH-CA and CH-PC PECs demonstrated higher porosity, which allows for increased water uptake and subsequent swelling.

#### 2.3.2. FTIR

**Figure 3:**
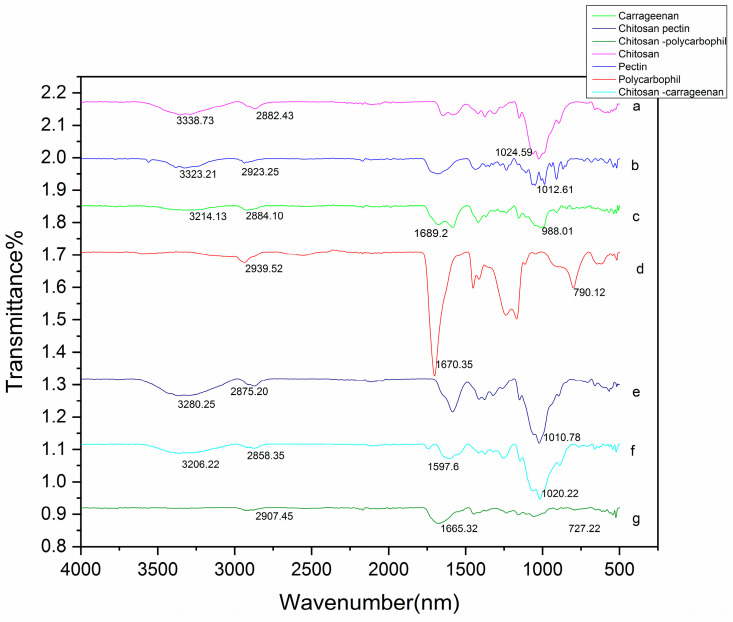
FTIR of a—chitosan, b—pectin, c—carrageenan, d—polycarbophil, e—chitosan–pectin, f—chitosan–carrageenan, g—chitosan–polycarbophil.

Fourier transform infrared spectroscopy was applied to evaluate the probable chemical interaction between polymers in the PEC (Figure 3). Examination of the chitosan spectrum reveals a strong absorption band attributed to the symmetrical bending vibration due to overlap of the -NH_2_ group and -OH group shown at 3338.73 cm^−1^; the absorption of the methylene CH group is visible at 2882.43 cm^−1^; the amine group and carbonyl group of the amino-glucose unit are visible at 1585.9 and 1646.8 cm^−1^, respectively. In pectin spectra, the carbonyl group and methyl ester group are displayed as a broad band at 1584.7 cm^−1^ and there is an aliphatic C-H group at 2923.25 cm^−1^ and an -OH group at 3323.21 cm^−1^. The carrageenan spectra show a -OH group at 3214.13 cm^−1^, an aliphatic C-H group at 2884.10 cm^−1^ and S=O broad bands of an SO_3_ group at 1235.7 and 1267.3 cm^−1^. The S-O bond at the C4 position in the galactose ring of carrageenan was displayed at 843.8 cm^−1^, and absorption bands at 908.1 cm^−1^ and 1014.3 cm^−1^ are assigned to the 3,6-anhydrogalactose unit [29]. Polycarbophil spectra displayed C=O stretching (hydrogen bonded) at 1701.6 cm^−1^, an aliphatic C-H group at 2939.53 cm^−1^, a weak band of COO^−^ symmetric stretching at 1451 cm^−1^ and the stretching bands of the C-O at 1237.8 and 1168 cm^−1^.

The PEC is the result of electrostatic interaction between polymers and its spectrum is the sum of the spectra of the individual polymers. There were changes observed in the spectra, such as band broadening, shifting of wave numbers and the appearance of new absorption bands that indicate polymer interaction during the formation of the PEC.

The CH-PE PEC spectrum indicates absorption peaks for the -OH group at 3280.25 cm^−1^ and there was a single broad absorption band at 1678.7 cm^−1^ masking the carbonyl group in pectin and the amide I and amide II bands of chitosan. This could be the result of interactions between the amino group of chitosan and the methylene group of pectin [31]. The CH-CA PEC spectra displayed an -OH group at 3206.22 cm^−1^, a new absorption band of NH_3_ groups at 1602.4 cm^−1^, and the displacement of the -S-O groups at 1020.22 cm^−1^. There was reduction in the sulphate group (S=O) peak intensity at 1254.4 cm^−1^. The weak absorption bands at 890 cm^−1^ and 1017.3 cm^−1^ could be assigned to an anhydrogalactose unit. These results are similar to those reported by Li et al. [32], confirming the interaction of the amino group of CH and the sulphate group of CA in the CH-CA PEC. CH-PC PEC spectra displayed a C-H group at 2907.45 cm^−1^ and symmetric COO- stretching at 1446.6 and 1367.1 cm^−1^. A new broad band at 1673.6 cm^−1^ could be attributed to the overlapping of COO^−^ and NH3^+^ in the PEC. This was consistent with the report by Pendekal et al. [33] and demonstrated the interaction between the amino group of chitosan and the carboxylic group of polycarbophil.

#### 2.3.3. DSC

**Figure 4:**
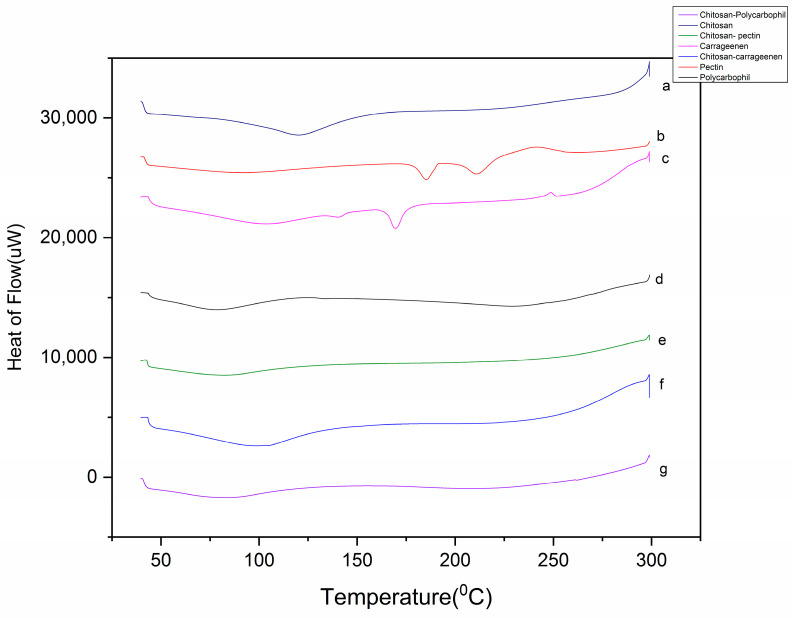
DSC of a—chitosan, b—pectin, c—carrageenan, d—polycarbophil, e—chitosan–pectin, f—chitosan–carrageenan, g—chitosan–polycarbophil.

A differential scanning calorimetry (DSC) study was performed (Figure 4) in order to study the changes in polymers during PEC formation. Chitosan displayed a broad endothermic peak at 119.9 °C that was associated with the evaporation of bound water, a glass transition at 240 °C and an exothermic peak beyond 300 °C related to the polymer degradation. Pectin demonstrated a very broad endotherm between 80 °C to 100 °C, indicating evaporation of free water trapped by the hydrophilic ends of the polymer, a sharp endothermic peak at 185 °C indicating the melting point and a broad endotherm at 210 °C indicating the thermal degradation of pectin [14]. Carrageenan displayed a very broad endotherm at around 100 °C, which represented the loss of water from the polymer. A small endotherm at 140 °C was attributed to the breaking of the sulfate bond in polymers, a sharp endothermic peak at 169 °C indicated the melting point of carrageenan and degradation of the polymer was observed above 280 °C [27]. Polycarbophil showed a broad endothermic peak around 78.5 °C representing the evaporation of the free water present in pectin due to hydrophilic groups. Near to 130 °C, a slight shift and change in heat capacity termed as a “z event” was observed, indicating glass transition, and from 200 °C onwards the spectrum showed polymer degradation [34].

The interaction between two polymers in a PEC can be often described by DSC. When polyelectrolyte complexes are formed, there is formation of electrostatic or weaker Van der Waal interactions between the two polymers. These adhesive interpolymer interactions may interfere with cohesive intra-polymer interactions between the same polymer chains, resulting in a loss of order or organization. This loss of order can be observed by formation of new endotherms or exotherms and shifting of the existing endotherm, as described in various research reports [35,36]. The DSC curve of the CH-PE PEC indicated an endothermic peak at 83.7 °C due to water evaporation, at around 240 °C it showed glass transition and after 260 °C the curve indicated polymer degradation. The disappearance of the peak of pectin at 185 °C confirmed the formation of a PEC. The CH-CA PEC curve showed a very broad endotherm starting from 60 °C and ending at 180 °C owing to loss of free and bound water and cleavage of interpolymer interactions. At around 240 °C glass transition was observed and from 260 °C onwards a broad endotherm represented degradation of the polymer. The sharp endotherm of carrageenan at 170 °C disappeared in the DSC curve and shifted and merged into a broad endotherm. This shifting of the endotherm and the appearance of the new endotherm is often an indication of the formation of a PEC [36]. The CH-PC PEC curve resulted in an endothermic peak at 82 °C due to the loss of free and bound water and a new very broad endothermic peak appeared from 150 to 280 °C, which indicated the breaking of electrostatic interactions between the polymers and initiation of polymer degradation in the PEC. The glass transition of chitosan was masked by this broad endotherm. There was complete a disappearance of the polycarbophil endotherm at 78 °C. This was in agreement with the report by Pendekal et al. [35] indicating the interaction between chitosan and polycarbophil.

### 2.4. In Vitro Mucoadhesive Study of PEC Using a Texture Analyzer

The efficacy of vaginal dosage forms is often limited due to their lower retention in the vagina. Mucoadhesive polymers interact with the vaginal mucosa through hydrogen bonds, ionic interactions and Van der Waal interactions and increase the retention time in vagina, which may further improve the local availability of drug. The mucoadhesion of polymers is improved by the formation of PEC owing to the increase in the number and density of mucoadhesive groups on the PEC structure that can establish contact with the mucosal surface. Luna et al. [36] described the mechanism of the mucoadhesion of PECs taking place in three steps; contact of polymer chains and mucous membrane, interpenetration of polymer chains in mucous layer by secondary forces of attraction and formation of stronger primary bonds between the polymer and mucosa resulting in stronger mucoadhesion. The mucoadhesive strength is often determined by observing the force required to separate the dosage form from the vaginal mucosa. Considering the similarity in mucoadhesion of gelatin discs and porcine vaginal mucosa, as reported by Emilia Szymańska et al. [37], gelatin discs were used in this study for comparing the mucoadhesion of PECs.

The mucoadhesion was greatly affected by the type of anionic polymer in the PEC (Table 1). The strength of mucoadhesion for PECs (CA, PE and PC) on gelatin discs was found to be 2.9 ± 1.2 g/cm^2^, 12.8 ± 0.8 g/cm^2^ and 2.4 ± 0.7 g/cm^2^, respectively, and the force of mucoadhesion was observed as 0.264 N, 1.255 N and 0.235 N, respectively. CH-PE PECs (*p* value 0.0056) demonstrated higher mucoadhesion compared to CH-CA PECs and CH-PC PECs.

In an acidic polymer, hydrogen bonding in the polymer due to -OH and -COOH groups and the open expanded conformation of the polymer in an ionized state are responsible for mucoadhesion. A higher degree of ionization may increase the ionic interaction with mucin. The degree of ionization of an acidic polymer increases in pH conditions greater than their pKa value. Considering the acidic vaginal pH, the mucoadhesive strength was determined at pH 4.8. At this pH, pectin (pKa 3.5) undergoes extensive ionization and hydrogen bonding, resulting in higher mucoadhesion with gelatin. The higher pKa values for CA (pKa 4.9) and polycarbophil (pKa 6.0) could have been responsible for their lower ionic interactions and mucoadhesion. Interestingly, Cazorla-Luna et al. [38] associated a lower degree of swelling in PECs to better mucoadhesion. Higher water uptake or swelling decreases the polymer mucoadhesion as a result of polymer–solvent interactions leading to reduced polymer chain mobility and penetration in the mucosa. This is in agreement with the observations of swelling and mucoadhesion for CH-PE PECs in the present study. The degree of swelling of CH-PE was found to be lowest among all PECs, resulting in the highest mucoadhesion.

#### 2.4.1. Antimicrobial Activity of PECs

All the PECs demonstrated good antifungal activity against *Candida albicans*. The activity was in the order: CH-PE > CH-CA > CH-PC. There were no significant differences (*p* > 0.1) in the antifungal activities of the PECs. The antifungal activity of the PECs could be attributed to presence of chitosan, which is reported to be effective against *Candida albicans* (Table 1). Chitosan, due to its cationic nature, is reported to interact with the negatively charged fungal membrane, thus altering the anion–cation balance and interfering with its integrity. Recently, Pei-Yu et al. [39] reported that chitosan inhibits components of the SAGA complex, which is responsible for fungal membrane integrity, and the MDR1 and CDR1 genes of the efflux transporter, leading to its antifungal activity. Although CA is also reported to have activity against *Candida* spp. [40], the CH-CA PEC did not show any increase in antifungal activity.

#### 2.4.2. Effect of Lyophilization on the Viability of *Lactobacillus casei*

The lyophilization process can have negative effects on the viability of probiotics within the PEC. The extreme cold temperatures and the formation of ice crystals during freezing can damage bacterial membranes, which may lead to decreased viability. Considering this, the viability of *L. casei* before and after lyophilization was assessed (Table 1). There was no significant negative effect of lyophilization on the viability of *Lactobacilli casei* evident from the data. The decrease in *L. casei* viability was by 0.3, 0.5 and 0.2 log CFU/mL when lyophilized with PECs of CH-CA, CH-PE and CH-PC, respectively. The polymers in PECs have the ability to entrap bacterial cells and act as non-permeating cryoprotectants that are responsible for inducing formation of ice in an amorphous form instead of crystalline, thus protecting the cell membrane from damage [41]. As the normal Lactobacilli cell count in vagina is 10^8^ cells per ml, the probiotic cell count in the PECs was found to be optimal.

### 2.5. Evaluation of PEC Hydrogel Inserts

The vaginal hydrogel inserts were in the weight range of 629.3 ± 0.95 to 631.2 ± 0.69 mg, indicating uniformity in the weight of the inserts. The thickness of the inserts was in the range 4.1 ± 2.04 to 4.6 ± 1.01 mm and the hardness was 6.5 ± 0.27 to 6.7 ± 0.29 kg/cm^2^. The drug content was observed to be from 90.5 ± 2.38 to 99.5 ± 1.42%.

**Figure 5:**
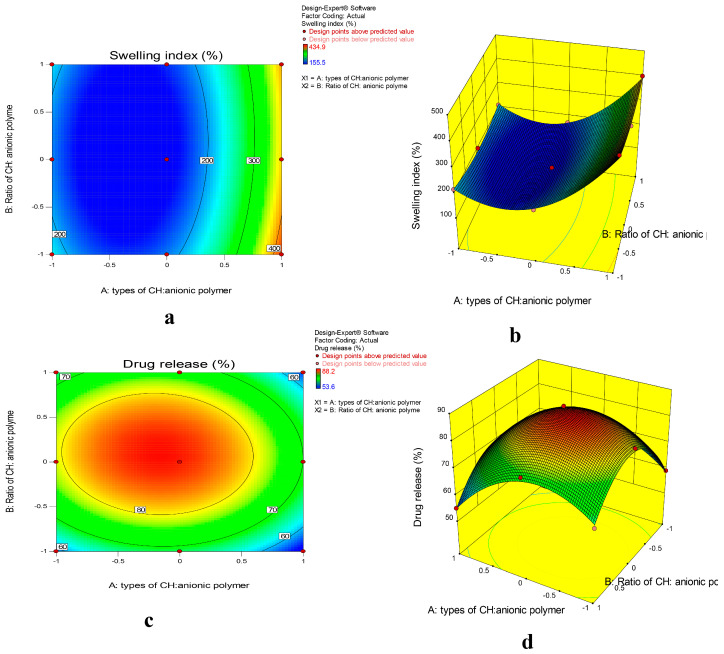
(**a**) Contour plot and (**b**) response surface graph showing the effect on swelling index. (**c**) Contour plot and (**d**) response surface graph showing the effect on drug release.

The effect of the type of anionic polymer (X_1_) used and ratio of chitosan:anionic polymer (X_2_) on the swelling of the PEC inserts (Y_1_) and the release of POS from the PEC inserts (Y_2_) was studied (Table 2 and Table 3). X_1_ was a categoric factor that was studied at 3 levels; carrageenan (−1), pectin (0) and polycarbophil (1), whereas X_2_ was a numeric factor that was studied at 3 levels, which were CH:anionic polymer ratio, 1:2 (−1), 1:1 (0) and 2:1 (1). The data were subjected to analysis of variance and multiple linear regression analysis to study the significance of the responses. The correlation between independent variables and their responses was obtained as polynomial equations (Equations (1) and (2)). Table 2 represents ANOVA data for responses Y_1_ and Y_2_. The surface response plots (Figure 5a,b) and contour plots (Figure 5c,d) were used to depict the relationship further.

#### 2.5.1. Effect on Swelling

The polymer swelling is an important phenomenon indicating the hydration of th polymer and is responsible for controlling the drug release.

Equation (1) indicates the effect of X_1_ and X_2_ on the swelling index (Y_1_).

Y_1_ = 149.07 + 96.38X_1_ − 9.06X_2_ − 6.6X_1_X_2_ + 135.18(X_1_)^2^ + 29.73(X_2_)^2^ (1)

The quadratic model was the best fit mathematical model for the swelling index. A *p* value lower than 0.05 (*p* value 0.0013) indicates the significance of the model. The closer values for the predicted R^2^ (0.9396) and adjusted R^2^ (0.9858) further confirmed the model’s significance. A high value for adequate precision (26.06) demonstrated the adequacy of the model to navigate design space. From the ANOVA data, it can be concluded that the type of anionic polymer (*p* value 0.0004) has a significant impact on the swelling of PEC-based inserts, whereas ratio of chitosan:anionic polymer did not show any significance (*p* value 0.1865). There was no significant interactive effect of the variables. The presence of the quadratic terms X_1_^2^ and X_2_^2^, in the equation implied non-linearity of the response.

All the hydrogel insert formulations were able to swell to a great extent in 4.8 pH citrate buffer. From the surface response plot and contour plot it is evident that the swelling of the CH-PC PEC inserts was highest, followed by the CH-CA and CH-PE inserts. Carrageenan is a linear polymer made from repeating units of D-galactopyranose. The negative charge on carrageenan is attributed to the presence of a sulfate group. During PEC formation, the sulfate group of carrageenan is mainly responsible for the interaction with chitosan. Pectin is a linear polysaccharide made from a galacturonic acid derivative. The galacturonic acid content and degree of methoxylation play a vital role in the swelling of this polymer. Polycarbophil is a highly cross-linked polymer of polyacrylic acid.

**Figure 6:**
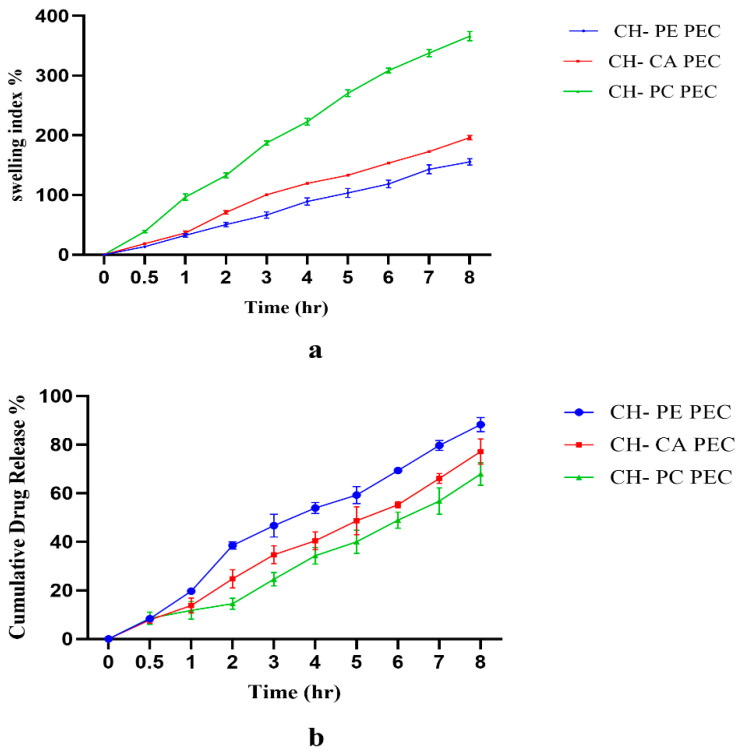
(**a**) Swelling behavior of PEC-based hydrogel inserts of posaconazole. (**b**) In vitro drug release of PEC inserts of posaconazole.

The swelling of a hydrogel matrix depends on the polymer and solvent interactions. Although interaction in terms of hydrogen bonding is important, osmotic and electrostatic interactions also play a major role. In the dry state, hydrogel matrices are in coiled structures that, upon water uptake, change into extended conformations. As the polymer extends, it forms hydrogen bonds with water that result in excessive swelling of the polymer. If the polymer is in ionized form, this will further help to create osmotic forces due to the difference in ion content inside and outside the hydrogel matrix resulting in additional water uptake or swelling. If the polymers are in ionized form, a similar charge on two neighboring polymer units will repel each other and create space for the uptake of additional water. These are the electrostatic interactions responsible for the swelling of a polymer. As the swelling study of the hydrogel inserts was performed in 4.8 pH citrate buffer, simulating the vaginal conditions, the degree of ionization of the polymer in that pH has affected the swelling to a great extent. As polycarbophil is a cross-linked polymer, the water uptake was highest in this PEC hydrogel. The water uptake was slightly higher in the CH-CA PEC than the CH-PE PEC. The low methoxyl content and low molecular weight of pectin could be responsible for its lower water uptake (Figure 6a).

The increase in interpolymer electrostatic interaction in PECs restricts the penetration of solvent into the polymer matrix, leading to lower swelling. The degree of swelling of PECs is not the summation of the swelling behavior of the individual polymer components and individual PEC polymers may show higher swelling than the interpolymer complex, according to the report by Carloza-Luna et al. [38]. Thus, the lower swelling index of the CH-PE PEC could also be indicative of higher ionic interactions between chitosan and pectin restricting the uptake of water. The degree of swelling of the inserts also depends on ratio of chitosan to anion in the PEC, concentration of the PEC in the inserts and other excipients added in the insert. It was observed that as the ratio of chitosan was increased in PECs, there was a decrease in the swelling index. This is in agreement with the report by Mosellanezhad et al. [42]. As chitosan is soluble in the swelling medium citrate buffer pH 4.8, the increase in the proportion of chitosan could have resulted in erosion of polymer matrix, resulting in a lower swelling index. After 8 h of swelling, the swelling index of the CH-PE PEC was found to be 155.7 ± 5.3%. Costa et al. [30] conducted a similar study for a CH-PE polyelectrolyte complex with montmorillonite clay, wherein the water uptake of the PEC in pH 5.8 buffer was found to be above 200%. However, in this work, the water uptake study was performed on a pure PEC and not on a PEC based on dosage form. Lu et al. [36] demonstrated that the addition of excipients to PECs is associated with mass loss that decreases the swelling index. Thus, the slightly lower water uptake of the CH-PE PEC insert in the present work could be attributed to the presence of excipients in the insert.

The swelling indices of the CH-CA and CH-PC PEC inserts were found to be 196.4 ± 3.7% and 366.1 ± 7.6%, respectively. Lu et al. [36] reported 500% water uptake after 8 h of swelling for their CH-PC PEC. However, in this work, the swelling study was conducted in water and using pure hydrogel. It has been well established that the pH of the swelling media has a direct impact on the swelling index, as it will determine the degree of dissociation of polymer at that pH. A higher degree of ionization in polymer chains allows repulsion of polymer chains, their solvation and subsequent swelling. However, the increased interaction of oppositely charged polymer chains will restrict the water uptake.

In order to further understand the kinetics and mechanism of swelling in PECs, the data were subjected to the Vergnaud model (1993) [43]. The Vergnaud model is expressed in the form of following equation.

$$Mt=K\times t^{n}$$

where *Mt* represents the amount of liquid transferred at time *t* and *K* is the swelling constant, which depends upon the amount of liquid transferred into the matrix after infinite time, the porosity of matrix and diffusivity. The exponent *n* indicates the mechanism of water uptake. The values of *K* and *n* can be obtained by plotting the log of percentage water uptake vs. log time [43,44].

Table 4 indicates the kinetic parameters for the Vergnaud model for the PECs of CH-PE, CH-CA and CH-PC prepared in a 1:1 ratio. A high value for the correlation coefficient (R^2^ > 0.98) indicated that the swelling kinetics can be well defined by this model. The swelling constant *K* indicates the rate of water uptake and therefore the rate of hydration of the polymer. This rate depends on the porosity of the matrix and the diffusivity of water into the matrix. The rate of polymer hydration depends on the presence of polar groups in the polymer structure and polymer–solvent interactions in the form of hydrogen bonds and ionic interactions. The rate of swelling or water uptake in the PEC inserts was in the order: CH-PC > CH-CA > CH-PE. The higher value for the swelling constant in the CH-PC PEC indicated higher water uptake and hydration of the polymer matrix resulting in sustained drug release from the swollen matrix.

The value of *n* in the equation indicates the mechanism of swelling. If value of *n* is less than 0.5, then the rate of diffusion of water in the matrix is slower than the polymer chain relaxation. Thus, the water diffusion rate will be the rate determining step for swelling. If the value of *n* equals 1, the rate of water penetration in the matrix is constant. Values of *n* between 0.5 and 1 indicate anomalous or complex behavior, wherein the rate of water diffusion equals to the polymer relaxation rate [43,44]. For all the PEC formulations, the *n* value suggests an anomalous or complex swelling mechanism for the PECs.

#### 2.5.2. Effect on Drug Release

Equation (2) indicates effect of X_1_ and X_2_ on posaconazole release from PEC hydrogel inserts after 8 h (Y_2_).

Y_2_ = 87.10 − 4.50X_1_ + 2.75X_2_ − 1.07X_1_X_2_ − 12.70(X_1_)^2^ − 16.55(X_2_)^2^ (2)

As indicated in the ANOVA data (Table 2), a *p* value less than 0.05 (*p* value 0.0082) implies the significance of the model. X_1_ and X_2_ both showed a significant impact on drug release (*p* values of 0.0225 and 0.0766, respectively). A high F value indicates the higher adequate precision; a lower difference between the predicted and adjusted R^2^ values further highlights the model significance. Both X_1_ and X_2_ have a non-linear effect on the response, as evident by the presence of quadratic terms. The type of polymer had a significant impact on POS release in the order: CH-PE > CH-CA > CH-PC PEC. The CH-PE PEC released 19.68 ± 1.2 of POS, the CH-CA PEC released 12.47 ± 1.5 and the CH-PC PEC released 11.78 ± 1.3 after one hour of dissolution, whereas the POS release after 8 h was 88.2 ± 2.4, 79.8 ± 3.04 and 67.9 ± 2.86%, respectively. These results are similar to the reports of drug release studies of PECs in the literature. Pendekal et al. [33] reported a 70% release of 5-fluorouracil from a PEC matrix of chitosan and polycarbophil when testing the drug release in 500 mL of simulated vaginal fluid at pH 4.2 for 8 h. Darvesh et al. [14] reported a 60 to 80% release of fluconazole from a chitosan–alginate-PEC-based insert when the dissolution was performed in 200 mL of phosphate buffer at pH 4.5. The drug release mechanism was Fickian diffusion.

The sustained drug release from the CH-PC matrix could be the result of its higher swelling compared with CH-PE and CH-CA PECs. This is in agreement with the results of the swelling index. When a hydrogel matrix comes in contact with a solvent, the coiled conformation of the polymer changes to an extended conformation, leading to increased water uptake as well as the swelling of the polymer. The drug present on the surface of the hydrogel matrix is released in a burst, whereas the drug present in the core of matrix slowly diffuses out upon the swelling of the polymer matrix. The degree of swelling will sustain the release further. In CH-PC, the swelling is high and the drug release is prolonged. The least swelling was observed in the CH-PE PEC, thus it released the drug at a faster rate than the other matrices (Figure 6b). The drug release was also affected by the ratio of chitosan to anionic polymer. Interestingly, the drug release was greatest at a CH-to-anion ratio of 1:1. When this ratio was changed to 2:1 or 1:2, the drug release was lower.

The drug release data were fitted into mathematical models, such as zero order, first order, Higuchi matrix, Korsmeyer–Peppas as well as Hixson–Crowell, to understand the drug release mechanism. The R^2^ value of the Korsmeyer–Peppas model was above 0.98, suggesting it as best fit model to describe the drug release mechanism. The K value of the Korsmeyer–Peppas model indicated the drug release rate. The value of K was in the order: CH-PE > CH-CA > CH-PC, indicating the highest drug release rate through the CH-PE matrix. This was in agreement with the release profile. The value of *n* for the Korsmeyer–Peppas model suggests the mechanism of drug release. If the *n* value is less than 0.5, the rate of drug diffusion through the polymer matrix is the slowest and the rate determining step. An *n* value of 0.9 indicates a constant drug release rate, where dissolution or erosion of polymer matrix is the rate determining step. Values of *n* from 0.5 to 0.9 suggest non-Fickian or anomalous release behavior, suggesting drug release due to a combination of the two mechanisms, drug diffusion through polymer matrix and subsequent polymer erosion. The *n* values of CH-CA, CH-PE and CH-PC were 0.69, 0.71 and 0.68, respectively. Thus, the drug release was the result of a combination of swelling, slow diffusion of the drug molecule from polymer matrix and slow dissolution or erosion of the polymer matrix due to excessive swelling.

Clinically, the drug release from the antifungal insert is very critical in order to maintain the concentration of drug above its minimum inhibitory concentration. As per the release profile, it is evident that 7 to 10 mg of POS was released per hour by the vaginal hydrogel insert matrix. As the MIC of POS is 0.007 to 2 µg/mL, the amount of POS released is sufficient to maintain its local tissue concentration above the MIC.

### 2.6. In Vitro Efficacy of the Formulations Using a Co-Culture Technique

**Figure 7:**
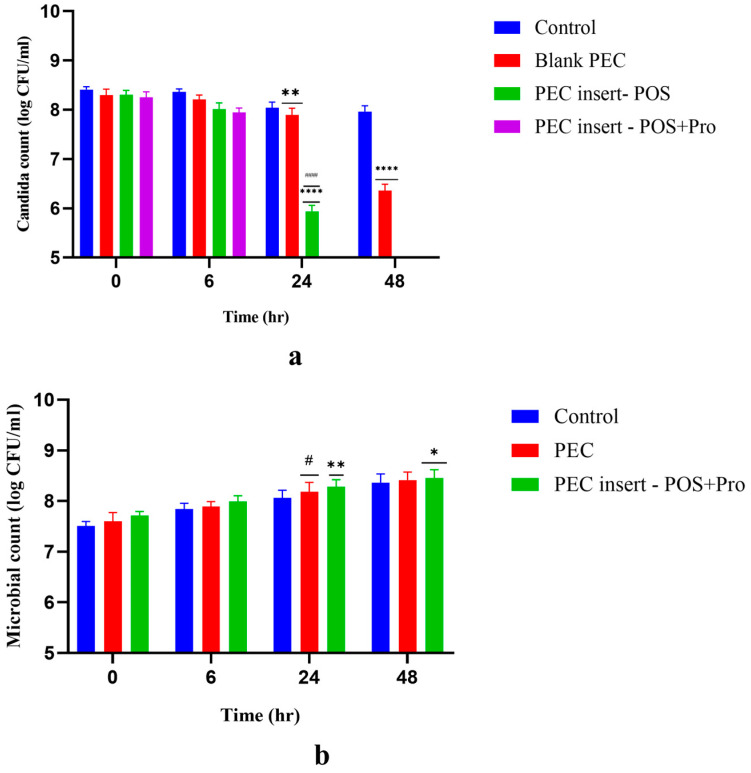
(**a**) Antimicrobial efficacy of the formulation (Candida count (log cfu/mL) of *Candida albicans* over the span of 0 h, 6 h, 24 h and 48 h). **** *p* value < 0.001, ** *p* value < 0.01, #### *p* value < 0.0001; (**b**) Microbial count (log cfu/mL) of *Lactobacillus casei* (0 h, 6 h, 24 h and 48 h) * *p* value < 0.01, ** *p* value < 0.001, # *p* value < 0.0001.

The performance of the developed CH-PE PEC was assessed using a co-culture test (Figure 7a). In this test, the effect of the formulation on growth of the probiotic *Lactobacillus casei* and the pathogen *Candida albicans* was studied over 0, 6, 24 and 48 h. The developed probiotic-loaded POS hydrogel insert will be effective if it supports the growth of *Lactobacilli* and decreases the pathogen count. Owing to the antifungal effect of chitosan, blank PEC inserts without drug showed a significant decrease (*p* < 0.01) in *Candida albicans* (2-log reduction) numbers after 48 h. The PEC inserts with posaconazole alone (group 3) or posaconazole and probiotic demonstrated a significant reduction (*p* < 0.001) in *Candida albicans* count after 24 h (5.93 and 4.77 log CFU/mL), whereas after 48 h there was a complete absence of *Candida albicans* growth. This indicated the significant antifungal efficacy of the developed hydrogel formulations. At 24 h, the PEC insert with posaconazole and probiotic resulted in a lower *Candida albicans* count than the PEC insert with posaconazole alone. This could be attributed to competitive inhibition of *Candida albicans* by *Lactobacillus casei* and also the ability of *Lactobacillus casei* to secrete antimicrobial substances. Probiotics are known to inhibit the growth of *Candida albicans* by secreting lactic acid, which reduces the pH of the vagina, making it not suitable for the growth of *Candida albicans*. Probiotics are also known to secrete hydrogen peroxide and bacteriocin as antimicrobial substances that further reduce the growth of candida. In one of our previous studies [45], the synbiotic effect of pectin as a prebiotic and *Lactobacillus casei* as a probiotic was studied on *C. albicans.* In vitro co-culture studies demonstrated a reduction in Candida albicans count from 6.81 log cfu/mL to 4.65 log cfu/mL (approximately a 2-log reduction) when cultured in the presence of *Lactobacillus casei* and pectin.

Figure 7b depicts the effect of the formulation on the growth of *Lactobacillus casei*. All the groups (control, PEC insert without *Lactobacillus casei* and posaconazole PEC insert with *Lactobacillus casei*) demonstrated similar growth for *Lactobacillus casei.* There was no inhibitory effect of posaconazole on *Lactobacillus casei*. This indicated that the *Lactobacillus casei* added in the formulation has ability to grow in favorable conditions without any inhibitory effect from other components of the PEC insert. There was an increase in *Lactobacillus casei* count by 0.8 log cfu/mL after 48 h of incubation with the PEC or PEC-based formulations. Thus, a stimulative effect of the formulation on *Lactobacillus casei* was observed. In our previous study [45], we demonstrated the effect of pectin as a prebiotic on lactobacilli from vaginal microflora in in vitro and in vivo models. The study demonstrated that pectin does not have any direct effect on pathogens; however, it has a stimulative effect on the lactobacilli of vaginal flora, such as *L. casei.* In vivo studies in a rat model also demonstrated the prophylactic and therapeutic effect of pectin in compromised flora. With pectin being a component of PEC insert, along with a probiotic, this would help in restoring the flora of an infected vagina that would otherwise be compromised. The restoration of *Lactobacilli* will further help in reducing the infection in the vagina.

## 3. Conclusions

A posaconazole hydrogel insert formulation was prepared using a polyelectrolyte complex combination using cationic chitosan with three different anionic polymers for vaginal delivery. PECs of chitosan with CA, PE and PC were prepared using a lyophilization technique and their formation was confirmed via SEM, FTIR and DSC analysis. The PECs demonstrated excellent mucoadhesion and antimicrobial activity that was attributed to chitosan. PEC-based hydrogel inserts of POS were optimized using a 3^2^ full-factorial design. The optimized formulation of inserts containing CH-PE PEC (1:1) demonstrated sustained drug release (88.2 ± 2.4%), excellent mucoadhesion (12.8 ± 0.8 g/cm^2^) and swelling after 8 h (155.5 ± 1.9%). Additionally, it demonstrated improved in vitro antifungal efficacy against *Candida albicans* in a co-culture test. The study revealed an increase in *Lactobacilli casei* and a drop in the viable count of *Candida albicans*, indicating that the formulation may be able to relieve symptoms while also assisting in the restoration of a healthy vaginal microbiota and preventing recurring vaginal infections. Thus, PEC-based hydrogel inserts could be a potential drug delivery system for the vaginal delivery of antifungal drugs such as posaconazole.

## 4. Materials and Methods

### 4.1. Materials

Posaconazole was obtained from Biocon Research Limited (Bangalore, India) as a gift sample. Kolliphor^®^ P 188 (P188) was received from BASF (Mumbai, India) as a gift sample. Chitosan (CH) (medium molecular weight, extra pure 90% DA) and pure pectin (PE) (low methoxyl content) were purchased from Srichem (Mumbai, India). Carrageenan sodium salt (CA) (Irish moss) was acquired from Himedia Ltd. (Mumbai, India). Noveon^®^ polycarbophil (PC) was received from Lubrizol Advanced Materials (Mumbai, India). Isopropyl alcohol, microcrystalline cellulose (MCC) and gelatin were purchased from LobaChemie (Mumbai, India). Sodium citrate, magnesium stearate, and talc were purchased from Research Lab (Mumbai, India). *Lactobacillus casei* (NCIM 5303) and *Candida albicans* (ATCC 10231) were used. De Man, Rogosa and Sharpe (MRS) agar, potato dextrose agar, and MRS broth were procured from Himedia (Mumbai, India). All other chemicals employed in the investigation were of analytical reagent (AR) grade.

### 4.2. Methods

#### 4.2.1. Preparation of Solid Dispersion

**Figure 8:**
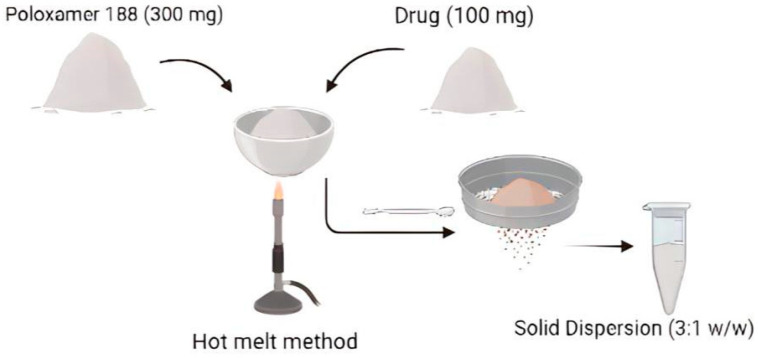
Systematic representation of solid dispersion.

Solid dispersions of POS and P 188 were prepared via the melt method in a 1:3 weight ratio (Figure 8). P 188 was melted at 80 ± 2 °C in a water bath and the POS was dispersed in the molten carrier with constant stirring. The dispersion was then cooled at room temperature and passed through sieve no 44 to obtain free-flowing powder [26]. The solid dispersion formation was confirmed by differential scanning calorimetry.

The saturation solubility of solid dispersion was determined in comparison with POS. For the determination of the solubility, an excess amount of POS and solid dispersion were added to 5 mL water and citrate buffer pH 4.8 separately in vials and then kept in an incubator shaker at 25 ± 2 °C and at 100 rpm (Remi CIS-18 Plus, Mumbai, India). Excess amounts of drug were centrifuged after 72 h and the supernatant was filtered using a 0.45 µ nylon membrane filter and then properly diluted and analyzed spectrophotometrically (Shimadzu UV-1800, Kyoto, Japan) at 260 nm [27].

#### 4.2.2. Preparation of Polyelectrolyte Complex (PEC)

For preparation of the PECs, CA, PE and PC were dissolved in distilled water at 2% *w/v* with continuous stirring and allowed to swell overnight. CH (2% *w*/*v*) was dissolved in acetate buffer pH 5. Each anionic polymer solution was added separately to chitosan solution with continuous overhead stirring for 2 h at 1000 rpm to obtain weight ratios of cation:anion of 1:2, 1:1 and 2:1. The prepared mixture was placed in the deep freezer for 24 h at −80 ± 2 °C (Remi ULT-185); this is the pre-freezing stage of lyophilization. The samples were then lyophilized (Martin Christ, Alpha 2-4 LSC, An der Unteren Sose, Germany) using the conditions of freezing for 30 min, warming up for 10 min, main drying for 4 h (vacuum 0.0050 m bar) and final drying for 3 h (vacuum 0.1000 m bar). The cycle was repeated for 2 days to remove any traces of residual moisture. The dried samples of PECs were collected. The lyophilized PECs were powdered, passed through sieve and characterized using SEM, FTIR and DSC. For preparation of the probiotic-loaded PECs, a standardized culture of *Lactobacillus casei* (1 × 10^9^ CFU/mL) was centrifuged at low temperature in a refrigerated centrifuge (Remi C-24 plus, Mumbai, India) at 5000 rpm, and the pellet was mixed with the PEC before freezing. The frozen samples were subjected to lyophilization using the same conditions as mentioned above [14].

#### 4.2.3. Formulation and Optimization of PEC-Based Hydrogel Inserts

Briefly, a probiotic-loaded PEC, a solid dispersion of POS, microcrystalline cellulose, sodium citrate, magnesium stearate and talc were mixed to obtain a uniform blend, and the mixture was compressed directly on a rotary tablet press (Rimek Mini Press MT-II, Ahmedabad, India) using a 12 mm punch.

The effect of the anionic polymer type in the PEC (X_1_) and the ratio of CH:anionic polymer in the PEC (X_2_) on performance of hydrogel inserts was studied using a 3^2^ full-factorial design. X_1_ is a categoric factor indicating the type of anionic polymer, CA (−1), PE (0) and PC (1). X_2_ is a numeric factor indicating the ratio of CH to each of the anionic polymers at three different levels, 1:2, 1:1 and 2:1. The primary impact of X_1_ and X_2_ on response variables, such as the swelling index after 8 h (Y_1_) and drug release after 8 h (Y_2_) was studied using Design-Expert^®^ software (version 11; Stat-Ease, Inc., Minneapolis, MN, USA) for statistical analysis. Table 5 describes the independent variables (X_1_ and X_2_) with their levels and responses (Y_1_ and Y_2_). Table 6 indicates the composition of the hydrogel vaginal inserts. To assess the significance of the examined components, analysis of variance (ANOVA) and linear regression analysis were used. The effect of the variables was further demonstrated using 3D surface and contour plots.

#### 4.2.4. Characterization of PEC

##### Morphological Study by FESEM

Field emission scanning electron microscopy (FESEM) (FEI, Nova Nano 450, Hillsboro, Oregon, USA) was used to determine the surface morphology of the PECs. In order to make the samples (5 mg) electrically conductive, a 20 nm layer of platinum was applied to them. In the FESEM chamber, the coated samples were scanned randomly by applying an acceleration voltage and a vacuum pressure of 10.00 KV and 1.30 × 10^−4^ pa, respectively. Different magnifications were used to produce the photomicrographs [46].

##### FTIR

FTIR absorption spectra for the polymers, CH, PE, CA and PC, and their PECs after lyophilization were recorded. The dry mixture of the sample and potassium bromide were placed in the sample holder and scanned from 400 to 4000 cm^−1^ using an FTIR spectrophotometer (Shimadzu, 8400S, Kyoto, Japan) [14].

##### Differential Scanning Calorimetry

The thermal analysis of the polymers CH, PE, CA and PC and their PECs was examined by differential scanning calorimetry (Hitachi, DSC7020, Tokyo, Japan). The analysis was carried out by heating the sample (1 mg) under a constant nitrogen flow rate of 20 mL/min and a heating rate of 10 °C/min. The thermograms were obtained in the temperature range 30–300 °C using a blank aluminum pan as a reference [46].

##### In Vitro Mucoadhesive Study

An in vitro mucoadhesion examination of PEC hydrogel inserts against a gelatin disc was performed using a texture analyzer (Ametek Brookfield CT3, Mumbai, India). The preparation of gelatin discs (20 mm in diameter, 2–3 mm in height) involved pouring a 30% (*w*/*w*) aqueous gelatin solution on a Petri plate and allowing jellification. The disc was mounted on the base of the texture analyzer and moistened with citrate buffer pH 4.8. Before testing, the insert was attached with adhesive tape to the other moving arm of the texture analyzer. In order to make contact with the mucosa for 10 s with 10 g of force, the arm holding the insert was lowered at a rate of 5 mms^−1^. The probe was then taken out of the mucosa. The force required to detach the insert from the mucosal membrane was recorded as the mucoadhesive force (N) [38].

##### In Vitro Antimicrobial Activity

The antimicrobial activity of the PECs was tested against *Candida albicans* using the agar well diffusion method. For *Candida albicans*, potato dextrose was used as a selective media sterilized in an autoclave (Meta-Lab, AI-7482, Mumbai, India) at 121 °C for 20 min. The sterilized media was poured into sterile Petri plates and kept for solidification under aseptic conditions. The plates were placed in a hot air oven (Bio-Technics AI-7982, Mumbai, India) for 30 min. The overnight cultures (0.1 mL) of *Candida albicans* were evenly distributed on agar media. The cups were cut using a cork borer of 6 mm diameter and 0.1 mL of PEC sample (12.5 mg of PEC in 10 mL) was added to each well. The samples were refrigerated for 30 min to enable diffusion. The plates were incubated at 37 ± 2 °C for 48 h in a bacteriological incubator (Bio-Techno Lab, Mumbai, India) and the zones of inhibition were measured [46].

##### Effect of Lyophilization on Viability of *Lactobacillus casei*

Probiotic-loaded lyophilized PECs (250 mg) were dispersed in 25 mL of MRS broth and serially diluted (10^−2^, 10^−4^, 10^−6^, 10^−8^) with same media before plating. The serial dilution samples were then plated on sterile MRS agar plates and the plates were incubated at 37 ± 2 °C for 24 h in a bacteriological incubator (Bio-Techno Lab, Mumbai, India). The resulting bacterial colonies were counted. The results were expressed in log CFU/mL as mean value and ±standard deviation.

#### 4.2.5. Characterization of PEC-Based Hydrogel Inserts

##### Average Weight, Hardness and Thickness

The average weight of the inserts was determined by weighing 20 inserts collectively and separately. The percent deviation in weight of the inserts was analyzed. Three inserts were chosen from each formulation batch and their thicknesses and hardnesses were measured using a digital tablet hardness tester (Labindia, TH1050 M, Navi Mumbai, India). The average thickness and the average hardness were then calculated.

##### Drug Content

The POS insert was crushed and dissolved in 250 mL of a methanol and water mixture. The resulting dispersion was shaken and ultrasonically agitated for an hour before filtering using a nylon membrane filter (0.45 µm). The filtrate was suitably diluted, and the absorbance of the filtrate was detected using a UV spectrophotometer at 261 nm (Shimadzu, UV-1800, Kyoto, Japan) [14].

##### Swelling Behavior

For observing the swelling behavior, a previously weighed insert (T1) was placed in a Petri dish containing 10 mL of citrate buffer pH 4.8. The insert was taken out of the dish at intervals of one hour, and its weight (T2) was recorded after the excess water that had been on its surface was removed. The following formula was used to determine the % water uptake:

$$Percent\;water\;Uptake=\frac{T2-T1}{T1}\times 100$$

T1 represents the insert’s initial weight and T2 is the insert’s final weight after swelling [44].

##### In Vitro Drug Release Study

USP apparatus II was used to carry out the study of in vitro dissolution (Veego, VDA-8D, Mumbai, India). An accurately weighed insert was placed at the bottom of a dissolution vessel filled with citrate buffer pH 4.8 and isopropyl alcohol in a 70:30 ratio (900 mL) as a dissolution medium, which was maintained at 37 ± 0.5 °C and 50 rpm. Aliquots of 10 mL each were withdrawn at different time intervals (0.5, 1, 2, 3, 4, 5, 6, 7 and 8 h). An equivalent volume of dissolution media was replaced immediately in order to maintain sink conditions. The concentration of the PEC was analyzed using a UV-visible spectrophotometer (Shimadzu, UV 1800, Kyoto, Japan) at 260 nm. The dissolution studies were carried out in triplicate. The data of drug release were fitted to different mathematical models to understand the mechanism of drug release [47].

##### In Vitro Efficacy of Formulation using a Co-Culture Technique

The in vitro efficacy of the POS hydrogel insert against *Candida albicans* was assessed using a co-culture technique. The efficacy of probiotic-loaded POS hydrogel inserts was compared with POS hydrogel inserts without a probiotic. The overnight standardized culture of *Candida albicans* was inoculated in MRS broth to a series of flasks; flask 1 was the control with *Candida albicans* and *Lactobacillus casei*, flask 2 was a blank PEC with *L. Casei* and *C. albicans* externally added, flask 3 contained the POS hydrogel insert without probiotic with *C. albicans* externally added and flask 4 contained the POS hydrogel insert with probiotic and with *C. albicans* externally added. These flasks were incubated for 48 h under aerobic conditions. At 0, 6, 24 and 48 h, 100 µL of the suspension was withdrawn from each flask, and serial dilutions (10^−2^, 10^−4^, 10^−6^, 10^−8^) were prepared using MRS broth. These dilutions (100 µL) were spread onto sterile agar plates and incubated at 37 ± 0.5 °C for 24 h in a bacteriological incubator (Bio-Techno Lab, India). MRS agar was used as the selective medium for the *Lactobacillus casei* and chloramphenicol yeast glucose agar for *Candida albicans*. Each plate of colonies was counted after 24 h and the viable count (CFU/mL) was determined using the formula given below [48].

$$CFU/mL=\frac{No\;of\;colonies\times dilution\;factor}{mL\;plated}$$

## Figures and Tables

**Table 1:** Evaluation parameters of PECs.

PEC Samples	Viability Count of PEC before Lyophilization (Log CFU/mL)	Viability Count of PEC after Lyophilization (Log CFU/mL)	Mucoadhesion Strength (g/cm^2^)	Inhibition Zone (mm)
CH-CA	8.3 ± 1.42	8.0 ± 0.78	2.9 ± 1.2	17.6 ± 0.57
CH-PE	8.2 ± 0.68	7.7 ± 1.4	12.8 ± 0.8	18.3 ± 0.68
CH-PC	8.4 ± 0.83	8.2 ± 1.12	2.4 ± 0.7	13.4 ± 0.62

**Table 2:** ANOVA responses for optimization of PEC hydrogel inserts.

Source	(Y1) Swelling Index			(Y2) Drug Release		
	Sum of Squares	F Value	*p*-Value	Sum of Squares	F Value	*p*-Value
			Prob > F			Prob > F
Model	94,723.16	111.83	0.0013	1041.88	32.39	0.0082
A—Types of CH–anionic polymers	55,738.48	329.02	0.0004	121.50	18.89	0.0225
B—ratio of CH:anionic polymer	493.23	2.91	0.1865	45.38	7.05	0.0766
AB	174.24	1.03	0.3852	4.62	0.72	0.4589
A^2^	36,549.07	215.75	0.0007	322.58	50.15	0.0058
B^2^	1768.14	10.44	0.0482	547.81	85.16	0.0027
Residual	508.22			19.30		
Cor Total	95,231.38			1061.18		

**Table 3:** Evaluation of PEC-based hydrogel inserts.

Batch	Hardness (kg/cm^2^)	Thickness (mm)	Drug Content (%)	Average Weight (mg)	Swelling Index (%) (8 h) (Y1)	Drug Release (%) (8 h) (Y2)
F1	6.6 ± 0.25	4.6 ± 1.01	99.5 ± 1.42	630.6 ± 1.42	217.6 ± 1.41	59.0 ± 2.87
F2	6.5 ± 0.27	4.2 ± 2.03	95.4 ± 2.57	631.1 ± 0.68	196.0 ± 3.07	79.8 ± 3.04
F3	6.6 ± 0.31	4.3 ± 1.02	91.3 ± 3.71	631.2 ± 0.69	209.5 ± 2.32	64.8 ± 1.89
F4	6.6 ± 0.24	4.2 ± 3.01	93.1 ± 1.32	630.0 ± 1.13	181.8 ± 1.84	65.4 ± 1.94
F5	6.7 ± 0.29	4.1 ± 2.04	99.5 ± 1.42	630.7 ± 0.77	155.5 ± 1.9	88.2 ± 2.4
F6	6.6 ± 0.26	4.4 ± 1.03	94.6 ± 1.52	629.8 ± 0.89	169.7 ± 2.28	74.6 ± 3.22
F7	6.5 ± 0.21	4.2 ± 2.01	92.2 ± 2.69	631.0 ± 0.71	434.9 ± 3.46	53.6 ± 2.68
F8	6.5 ± 0.28	4.3 ± 3.02	97.3 ± 3.27	630.4 ± 1.02	366.1 ± 2.53	67.9 ± 2.86
F9	6.6 ± 0.23	4.1 ± 5.04	90.5 ± 2.38	629.3 ± 0.95	400.4 ± 3.1	55.1 ± 2.5

**Table 4:** Kinetic parameters for the Vergnaud model of the swelling of PECs and Korsmeyer–Peppas model for drug release.

PEC Type	Vergnaud Model			Korsmeyer–Peppas Model		
	R^2^	*K*	N	R^2^	*K*	*n*
CH-CA	0.991	1.63	0.77	0.992	16.81	0.69
CH-PE	0.994	1.42	0.75	0.993	21.08	0.72
CH-PC	0.992	5.80	0.67	0.989	14.39	0.68

**Table 5:** Independent variables and responses in 3^2^ full-factorial design.

**Levels**	**Independent Variables**		**Responses**	
	**X1**	**X2**	**Y1**	**Y2**
	**Types of Anionic Polymer**	**Ratio of Chitosan:Anionic Polymer**	Swelling Index after 8 h	Drug Release after 8 h
−1	Carrageenan	1:2		
0	Pectin	1:1		
1	Polycarbophil	2:1		

**Table 6:** Composition of hydrogel vaginal inserts.

Ingredient (mg)	F1	F2	F3	F4	F5	F6	F7	F8	F9
CH:CA (1:2)	250								
CH:CA (1:1)		250							
CH: CA (2:1)			250						
CH:PE (1:2)				250					
CH:PE (1:1)					250				
CH: PE (2:1)						250			
CH: POLY (1:2)							250		
CH: POLY (1:1)								250	
CH: POLY (2:1)									250
Solid Dispersion	200	200	200	200	200	200	200	200	200
Magnesium Stearate	6	6	6	6	6	6	6	6	6
Microcrystalline cellulose	18	18	18	18	18	18	18	18	18
Sodium monocitrate	150	150	150	150	150	150	150	150	150
Talc	6	6	6	6	6	6	6	6	6

Average weight of each insert is 630 mg.

## Data Availability

Data used to support the finding is available on demand from corresponding authors on request. The data are not publicly available due to ongoing research using a part of the data.
